# Synergistic Inhibition of MRSA by Chenodeoxycholic Acid and Carbapenem Antibiotics

**DOI:** 10.3390/antibiotics12010071

**Published:** 2022-12-31

**Authors:** Kaiyu Cui, Weifeng Yang, Shuang Liu, Dongying Li, Lu Li, Xing Ren, Yanan Sun, Gaiying He, Shuhua Ma, Jidan Zhang, Qing Wei, Yi Wang

**Affiliations:** 1 Experimental Research Center, China Academy of Chinese Medical Sciences, Beijing 100700, China; 2 Nanchang Institute of Technology, Nanchang 330044, China

**Keywords:** chenodeoxycholic acid, MRSA, carbapenems, penicillin-binding proteins, synergistic inhibition

## Abstract

Methicillin-resistant *Staphylococcus aureus* (MRSA) has posed a severe global health threat. In this study, we screened an antibiotic and non-antibiotic combination that provides a viable strategy to solve this issue by broadening the antimicrobial spectrum. We found that chenodeoxycholic acid (CDCA) could synergistically act with carbapenem antibiotics to eradicate MRSA-related infections. This synergy specifically targets MRSA and was also validated using 25 clinical MRSA strains using time–kill analysis. We speculated that the underlying mechanism was associated with the interaction of penicillin-binding proteins (PBPs). As a result, the synergistic efficiency of CDCA with carbapenems targeting PBP1 was better than that of β-lactams targeting PBPs. Moreover, we showed that CDCA did not affect the expression level of PBPs, but sensitized MRSA to carbapenems by disrupting the cell membrane. In our study, we have revealed a novel synergistic combination of antibiotics and non-antibiotics to combat potential bacterial infections.

## 1. Introduction

*Staphylococcus aureus* is a major human pathogen that causes a variety of clinical infectious diseases [1,2]. The most common sites of *S. aureus* infection are skin and soft tissues, and severe infections include bacteremia, pneumonia, endocarditis, osteoarthritis, and toxic shock syndrome [3,4,5]. The mortality rate of patients with *S. aureus* bacteremia in the “pre-antibiotic era” exceeded 80%, which was improved by the discovery of penicillin. However, bacteria could develop resistance to penicillin by expressing β-lactamases [6]. Methicillin-resistant *S. aureus* (MRSA) can develop resistance through the horizontal transfer of the SCCmec genetic element, which is a complex set of genes that carries the *mecA* gene encoding a penicillin-binding protein (PBP2a) with a lower affinity for β-lactamase [7]. Except for the latest fifth-generation cephalosporins, such as ceftaroline, MRSA showed resistance to almost all β-lactams. MRSA also developed resistance to other types of antibiotics, including vancomycin, linezolid, and daptomycin (DAP) [8,9,10], which are considered the last resort for the treatment of severe MRSA infections, further complicating the treatment of MRSA [11,12]. Given the increase in the number of multi-drug resistant MRSA strains and the slowdown in the antimicrobial drug development process, we urgently need new alternative treatments to fight against bacterial infections.

Carbapenems have been shown to have a broader antibacterial spectrum than existing β-lactams [13]. Carbapenems with different antimicrobial activities, including imipenem, panipenem, and doripenem, are effective against Gram-positive bacteria, while meropenem, biapenem, ertapenem, and doripenem have certain therapeutic effects on Gram-negative bacteria [14]. Additionally, carbapenem antibiotics showed better stability against β-lactamase when compared to other β-lactams [14]. However, except for tomopenem, other carbapenems showed a limited inhibitory effect on MRSA [13].

Bile acids, at concentrations higher than their critical micelle concentration, are powerful detergents capable of causing membrane dissolution through the formation of mixed micelles [15]. This rapid dissolution leads to leakage of cell content and cell death [16]. Bile acids can interact with the phospholipid bilayer of membranes through hydrophobic interactions to increase membrane fluidity and permeability [16,17,18]. Although bile acids have broad-spectrum antibacterial activity against Gram-positive pathogens, the concentration required for bactericidal activity is cytotoxic to host cells.

Our previous studies revealed that in traditional Chinese medicine (TCM), Tanreqing (TRQ), a Chinese herbal preparation extracted from *Scutellariae radix* (Huang Qin), *Lonicerae flos* (Jin Yin Hua), *Forsythiae fructus* (Lian Qiao), *Ursi fel* (Xiong Dan), and *Naemorhedi cornu* (Shan Yang Jiao), could effectively combat infections associated with *Pseudomonas aeruginosa* and *S. aureus* [19,20,21,22]. However, the mode of action of TRQ is not fully understood with respect to antimicrobial activity.

In this study, our aim was to evaluate the effect of subcytotoxic concentrations of chenodeoxycholic acid (CDCA), an effective component of TRQ, to examine whether it could be used in synergy with carbapenem to efficiently eradicate MRSA. We ultimately speculated a method targeting membrane function with small molecules to overcome carbapenem resistance in MRSA infection and other clinically relevant pathogens.

## 2. Materials and Methods

### 2.1. Strains and Culture Conditions

The bacterial strains used in this study are listed in Table S1. Strains were grown under standard culturing conditions at 37 °C in either Mueller–Hinton broth (MHB, Oxoid) or Lysogeny broth (LB, Oxoid). The clinical isolates were collected from the clinical laboratory of Dongzhimen Hospital and Guanganmen Hospital, Beijing, China.

### 2.2. Minimum Inhibitory Concentration (MIC) Assays

All antibiotics and bile acids were purchased from the China National Institutes for Drug Control. MICs were determined using the broth dilution assays, as described previously [20]. Briefly, 5 × 10^5^ CFU/mL were incubated with varying concentrations of antibiotics in a total volume of 200 μL of MHB in a 96-well plate. MICs were determined after incubation at 37 °C for 18~24 h.

### 2.3. Checkerboard Synergy Assays

A checkerboard broth dilution assay was performed to calculate the fractional inhibition concentration index (FICI). A total of 5 × 10^5^ CFU/mL was incubated in a 96-well plate that was distributed to an 8 × 8 matrix by 2-fold serial dilutions of each compound added to 2-fold carbapenem dilutions. The FICI was calculated as follows: FICI = (MIC of compound A in combination/MIC compound A alone) + (MIC compound B in combination/MIC compound B alone). A FICI ≤ 0.5 was interpreted as synergy, a 0.5 < FICI ≤ 1 indicates an additive effect, a 1 < FICI ≤ 2 represents no interaction, and a 2 < FICI represents antagonism.

### 2.4. Time–Kill Curve Assay

Time–kill analysis was used to determine *S. aureus* growth in the presence of various antibiotics, used as monotherapies or in combination. Where indicated, a bacterial suspension was added to each solution (5 × 10^6^ CFU/mL, 5 mL) and incubated at 37 °C under shaking. The growth control flasks contained only bacteria and 5 mL of MH broth. Each aliquot was serially diluted, seeded on MH agar plates, and incubated for 24 h at 37 °C in order to determine the number of CFU/mL. The colonies were counted only on plates that exhibited 30 to 300 colonies. Subsequently, time–kill curves were generated by plotting log10 CFU/mL against time (h).

### 2.5. Structured Illumination Microscopy (SIM)

*S. aureus* cultures grown at 37 °C in LB medium were diluted to 1:1000 in fresh LB medium and grown until the mid-logarithmic phase for 5 h at 37 °C. Subsequently, cells were treated, with or without CDCA, for 30 min at 37 °C. The cells were then stained with 2 μg/mL wheat germ agglutinin conjugate (WGA-488, Invitrogen, Waltham, MA, USA) at 37 °C under agitation for 10 min. Unbound dye was removed from the media by washing the cells with PBS, and the cells were then incubated with Nile Red (10 mg/L) for 10 min at room temperature and placed on an agarose pad containing 50% LB in PBS. For structured illumination microscopy, cells were viewed using a DeltaVision OMX (Applied Precision/GE Healthcare, Parramatta, Australia) comprising an OMX optical microscope (version 3), using a 561 nm laser for Nile Red, 488 nm laser for WGA-488, and 100 ms exposure.

### 2.6. Proteomic Analysis

The proteomic analysis of the bacterial samples was carried out according to methods previously described, with minor modifications [23].

## 3. Results

### 3.1. CDCA Sensitizes MRSA to Carbapenem Killing

**Figure 1:**
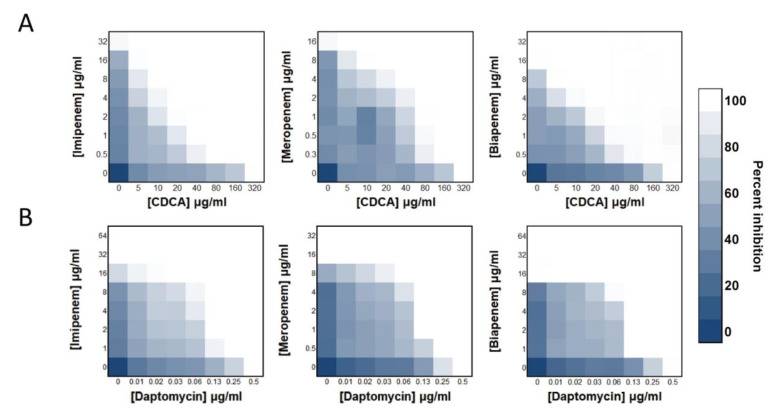
CDCA sensitizes MRSA to carbapenem killing. (**A**) Synergy between CDCA and carbapenems, evaluated against MRSA ATCC 43300. (**B**) Synergy between daptomycin and carbapenem, evaluated against MRSA ATCC 43300.

We found that cholic acid substances can sensitize *S. aureus* to carbapenem antibiotics. The synergistic efficiency of CDCA with carbapenems (FICI: 0.1875~0.125; Figure 1A) was higher than that of DAP with carbapenems (FICI: 0.3125; Figure 1B). Specifically, 1/16 MIC of CDCA (20 μg/mL) and 1/16 imipenem effectively repressed the growth of MRSA. The synergistic efficiency of carbapenem imipenem with CDCA was better than that of meropenem and biapenem. Among them, CDCA demonstrated a FICI of 0.125 for imipenem and 0.1875 for the rest of the carbapenems tested. The most prominent finding was that the combination of CDCA and imipenem had a strong synergistic inhibitory effect on MRSA, which was specific to MRSA. The drug combination did not have an effect on Gram-negative bacteria, and its effect on Gram-positive bacteria was similar to that of *S. aureus* (Figure S1), neither of which could achieve the synergistic efficiency shown in MRSA.

### 3.2. CDCA Improves the Bactericidal Effect of Antibiotics Targeting PBP1 and PBP2

**Figure 2:**
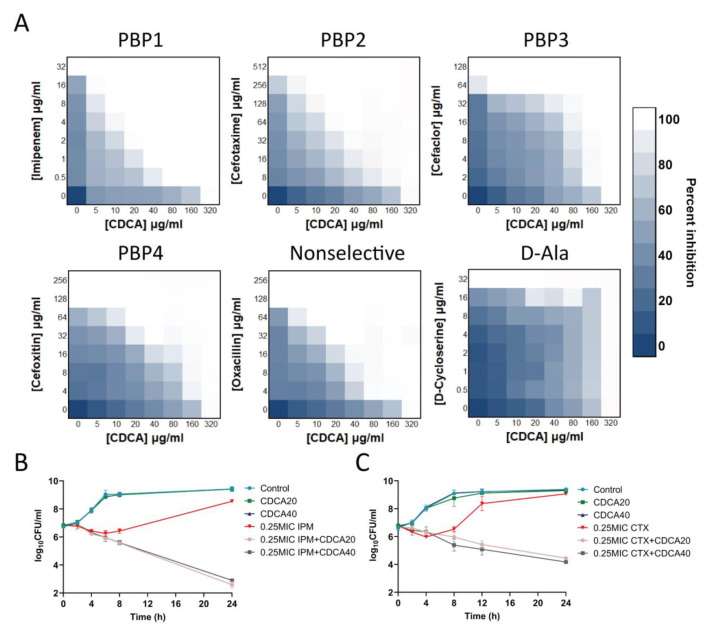
Combination of CDCA with a variety of cell wall-targeting antibiotics. (**A**) The combination of CDCA and cell wall-targeting antibiotics, include imipenem (PBP1 selective), cefotaxime (PBP2 selective), cefaclor (PBP3 selective), cefoxitin (PBP4 selective), oxacillin (non-selective PBPs), and D-cycloserine (D-Ala). (**B**) MRSA ATCC 43300 was grown to the mid-logarithmic phase and challenged with 0.25 × MIC (8 μg/mL) of imipenem, with or without CDCA (20 μg/mL or 40 μg/mL). The survivors were enumerated at the indicated time points. All experiments were carried out in biological triplicate. The error bar represents the mean ± SEM. (**C**) MRSA ATCC 43300 was grown to mid-logarithmic phase and challenged with 0.25 × MIC (128 μg/mL) of cefotaxime, with or without CDCA (20 μg/mL or 40 μg/mL). The survivors were enumerated at the indicated time points. All experiments were carried out in biological triplicate. The error bar represents the mean ± SEM.

We found that CDCA had synergistic effects with β-lactam drugs that act on the cell wall. Among them, the combination of antibiotics aimed at PBP1 showed the best synergistic effect: 1/16 MIC CDCA (20 μg/mL) and 1/16 MIC imipenem effectively inhibited the growth of MRSA, and the FICI of cefotaxime targeting PBP2 was 0.1875. The synergistic effect of oxacillin that did not target specific PBP, but acted on PBP2, was superior to that of cefaclor targeting PBP3 and cefoxitin targeting PBP4 (Figure 2A). However, CDCA did not act synergistically with other non-β-lactam antibiotics that also act on cell walls, such as D-cycloserine, which acts on D-Ala. We further demonstrated that the combination of CDCA and carbapenems, including imipenem, meropenem, and biapenem, was effective against all 25 clinical strains, including MU50. FIC values were distributed in a range from 0.09375 to 0.75 (Table 1), indicating a synergistic effect. The analysis of the killing curve showed that CDCA sensitized MRSA to imipenem (6 logs) and cefotaxime (4 logs; Figure 2B). It should be noted that CDCA (20 μg/mL) could significantly inhibit MRSA in the presence of imipenem (0.25 MIC), while MRSA growth with imipenem alone (0.25 MIC) reverted to the control level (>10^8^ CFU/mL).

### 3.3. Proteome Analysis Revealed That CDCA Perturbed the Bacterial Cytoplasmic Membrane

**Figure 3:**
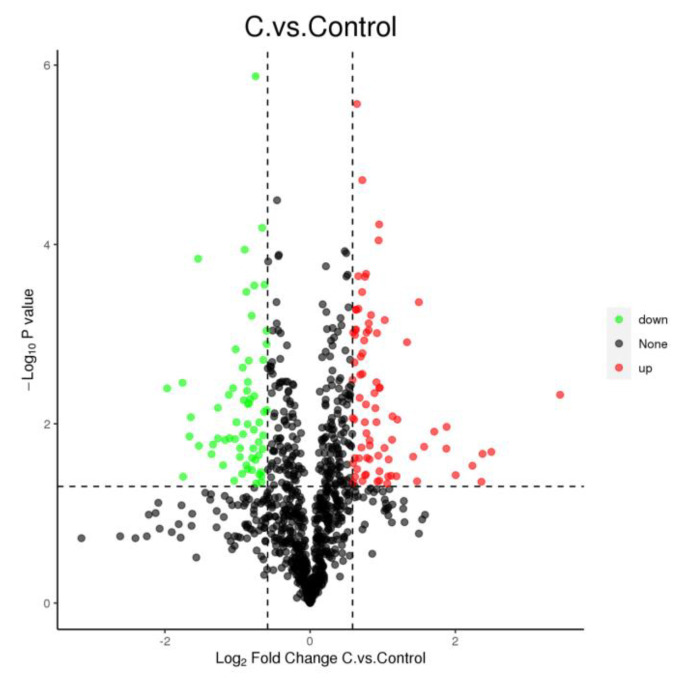
Volcano diagram of differential proteins in *Staphylococcus aureus* cells after CDCA treatment; down—negatively regulated; up—positively regulated; none—not significant.

The CDCA treatment affected the expression of 144 proteins (Figure 3), where 81 proteins were upregulated (*p* < 0.05), and 63 proteins were downregulated (*p* < 0.05). The functional classification of these proteins with altered expression revealed significant changes in proteins involved in cellular structure, virulence, amino acid transfer, and metabolism at the translational level in CDCA-treated cells.

Specifically, as seen in Table S2, the proteins involved in cellular structure were found to be affected. First, various proteins related to cell wall synthesis were upregulated, such as UDP-N-acetylglucosamine 1-carboxyvinyltransferase 1 and 2 by *murA1* and *murA2*; *murD* expressing UDP-*N*-acetylmuramoylalanine-D-glutamate ligase; and phospho-*N*-acetylmuramoyl-pentapeptide-transferase by *mraY* to antagonize CDCA perturbation on the bacterial envelope. Second, *recU* encoding PBP-associated factor A was found to be positively regulated, suggesting that the role of CDCA may be related to the abnormal division of MRSA and therefore, the abnormal function of the PBP protein.

**Figure 4:**
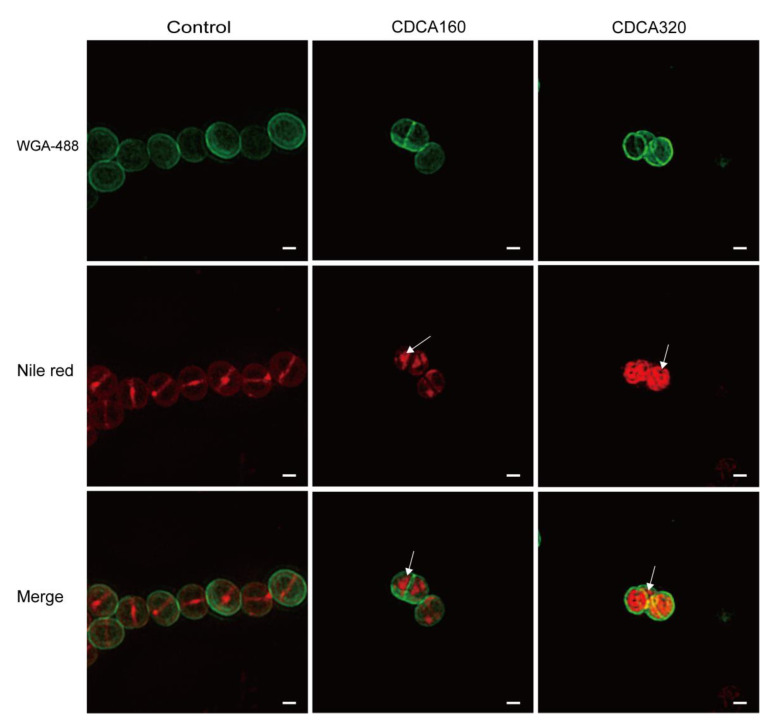
CDCA altered the bacterial cytoplasmic membrane. Microscopy analysis of CDCA-treated MRSA cells. The cells were labeled with green fluorescence (WGA-488) indicating the cell wall, and red fluorescence (Nile red) indicating the cell membrane and the septum. Arrows indicate an abnormal membrane topology. The scale bar is 200 nm.

To explore the effect of CDCA on the structure of the bacterial envelope, structured illumination microscopy (SIM) was adopted, and the results showed that cells in the blank control group formed a complete circular division diaphragm on the central surface of the cell, enabling bacteria to undergo normal contraction division. Vesicles were observed on the cell membrane in the CDCA group (160 μg/mL), and in the high concentration CDCA group (320 μg/mL), the cell membrane showed more abnormal structure (Figure 4). In conclusion, CDCA disrupted the normal function of PBPs on the cell membrane by disrupting the membrane and enhanced the killing effect of β-lactams targeting PBPs.

## 4. Discussion

Our previous study found that TRQ, a traditional Chinese medicine, had an antibacterial effect on MRSA and could interfere with the division of *S. aureus* [18]. In this study, we considered the cholic acid component CDCA as the main factor to investigate its inhibitory effect on cell division, and we focused on its synergistic effect with cell wall-targeting antibiotics. We chose a positive control, DAP, the most commonly used membrane damage agent, which has become the first-line drug for the treatment of challenging infections caused by MRSA and vancomycin-resistant *Enterococcus faecium* [24,25,26], which was reported to enhance the bactericidal activity of β-lactams. Moreover, carbapenems specifically targeting PBP1 have been shown to have a higher synergistic efficiency with DAP than with β-lactams bound to PBP1-4 [27]. The reason was that the *pbpA* gene that encodes PBP1 is a part of the cell wall/cell division gene cluster [28], which could be induced to increase in the DAP-induced membrane damage [29]. Subsequently, we found that the synergistic efficiency of carbapenems with CDCA was much higher than that of DAP. The compensatory cellular response to CDCA was to increase the septum and the site of cell division, similar to that of DAP, and this stress response likely made cells particularly sensitive to PBP1-targeting carbapenems. However, in line with previous data obtained from transcriptomics (**GSE193011**), the five PBPs (PBP1, PBP2, PBP2a, PBP3, PBP4) in MRSA existed after CDCA treatment, but did not show significant differences in gene expression and protein level (Table S2). Therefore, CDCA may not cooperate with β-lactams by affecting the expression level of PBP. Furthermore, it was speculated that CDCA may alter the normal function of the bacterial envelope, including the cell wall and cell membrane of MRSA, thus disrupting the function of PBP on the cell membrane and enhancing the killing effect of antibiotics targeting PBPs.

Two essential PBPs are involved in the peptide synthesis of *S. aureus*: PBP1 has a functional transpeptidase domain, which is mainly responsible for initiating cell division [30], and PBP2 is a bifunctional transglycosylase-transpeptidase [31]. The *mecA* gene that encodes a new PBP, PBP2a, could allow MRSA to proliferate, even in the presence of β-lactams. In MRSA, the synthesis of a new cell wall peptidoglycan requires the interaction of PBP2 with SCCmec encoded PBP2a, PBP2, and PBP2a, which serve as transglycosylase and cross-linked transpeptidase, respectively [32]. Therefore, PBP2a dependence is a “lethal weakness” of MRSA in cell wall peptidoglycan biosynthesis. Compounds that interfere with membrane function could synergize with β-lactam against MRSA. The function of the membrane appears to be strongly related to bacterial division [33], and we observed that the septum was widely distributed in the cells rather than concentrated in the center of division after treatment with bile acid. PBP1, PBP2, PBP3, and PBP4 are colocalized in the septum and the site of cell division [34]; contrastingly, PBP2a localized in lipid rafts on the membrane [35]. This different localization may be the reason why the synergistic effect of CDCA with β-lactams was specific for MRSA.

In proteomics analysis, we did not explicitly observe a difference in PBPs levels, but we detected an increase in RecU. In *S. aureus*, RecU is encoded in the same operon as PBP2, and during normal cell division, RecU and PBP2 are not co-expressed from the same operon; additionally, RecU is required for correct chromosome segregation and repair of DNA damage [36]. Elevated RecU values represented the self-protection of *S. aureus* upon CDCA treatment, which may disrupt normal proper cell division. This was consistent with our previous report that TRQ caused abnormal bacterial division, including increased activity of FtsZ GTPase, decreased autolysin, and strong inhibition of the expression of FtsZ, FtsW, and other proteins related to bacterial division [19]. The resulting increase in the division sites and septum is an effective means of combating cell membrane damage. The rise in FtsZ GTPase activity also indirectly indicated that bile acid may not be an inhibitor of FtsZ, and that the inhibition of division is only a compensatory behavior caused by membrane damage. This is because the inhibition of cell division is critical for survival under DNA damage, which induces bacterial SOS responses that inhibit cell division during repair [37,38,39]. The defects in the formation of the cell wall and the membrane indicated the occurrence of the relocalization of proteins involved in cell division and cell wall synthesis, and it was likely that membrane perturbation destroyed the normal cooperation of PBP2a with PBP2, leading to strong synergy between CDCA and antibiotics targeting PBP2.

Glycopeptides (GP), lipopeptides (LP), and lipoglycopeptides (LGP) are important antimicrobial agents targeting the envelope for the treatment of invasive MRSA infections. Studies have shown that if MRSA’s sensitivity to beta-lactam increases, it decreases its sensitivity to LP (i.e.， the “seesaw effect”) [40,41]. For example, failure of DAP treatment is usually due to the acquisition of a functional acquisition mutation affecting the MprF protein, leading to the failure of PsrA expression, which is instead sensitive to β-lactam targeting PBP2. However, the mechanism of CDCA resistance remains unclear. We tested 25 clinical MRSA strains with different carbapenem sensitivity, but their MIC values for CDCA were all 320 μg/mL, and no similar “seesaw effect” was observed. In the future, we will try to unravel the underlying mechanisms.

Bile acids and their derivatives have several non-negligible advantages over other available anti-MRSA drugs. First, selective resistance to bile acids is more difficult to evolve. This occurs because of the bacteria trying to modify the cell envelope to avoid being a drug target, which depends on the strategies of increasing the division sites and intervals, and this will lead to improper growth. In the future, we will accumulate more experimental data to prove this hypothesis. Second, these antibiotics targeting the membrane often cooperate with other antibiotics with different targets. However, the limitation of these drugs is mainly due to the potential toxicity problem, which is related to the inadequate selection of lipid components from bacterial cell membranes.

## Figures and Tables

**Table 1:** Distribution of FICI by CDCA combined with carbapenem in 25 MRSA strains.

	FICI								
	0.09375	0.125	0.1875	0.25	0.3125	0.375	0.5	0.625	0.75
IPM	1	1	6	4	1	5	0	1	6
MEM	0	0	2	7	3	5	1	2	5
BPM	0	1	5	7	1	3	1	0	7

## Data Availability

Not applicable.

## Supplementary Materials

The following supporting information can be downloaded at: https://www.mdpi.com/article/10.3390/antibiotics12010071/s1. Figure S1: CDCA combined with imipenem against common pathogens. Table S1: Strains used in this study. Table S2: CDCA-treated MRSA differential proteins in exponential growth phase.
