# Supplementary material for: Synergistic Inhibition of MRSA by Chenodeoxycholic Acid and Carbapenem Antibiotics
Source: Antibiotics (Basel). 2022 Dec 31;12(1):71. doi: 10.3390/antibiotics12010071 (PMC9854648; doi:10.3390/antibiotics12010071)
Supplement: Supplementary file 1 [file antibiotics-12-00071-s001.zip › antibiotics-2032840-supplementary.pdf]

Supplementary Information for

Synergistic inhibition of MRSA by chenodeoxycholic acid and carbapenem antibiotics

Kaiyu Cui[1†], Weifeng Yang[1†], Shuang Liu[1], Dongying Li[1], Lu Li[1],Xing Ren[1], Yanan Sun[1], Gaiying He[1], Shuhua Ma[1], Jidan Zhang[1], Qing Wei[2*], Yi Wang[1*]

[*]Corresponding author: Email: vubwqing@hotmail.com (Q.W.) and prof.wangyi@foxmail.com (Y.W.).
[†]These authors have contributed equally to this work.

This PDF file includes:

Supplementary Text
Figure S1#
Tables S1 and S2#

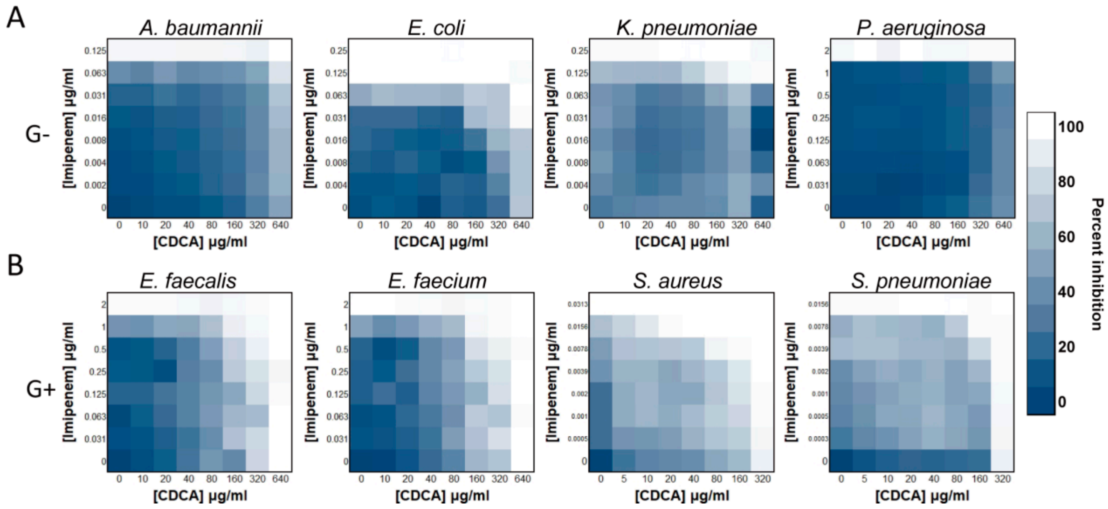

Figure S1. CDCA combined with imipenem against common pathogens.

(A) The combination of CDCA and imipenem, evaluated against four G^- bacteria, *Acinetobacter baumannii*, *Escherichia coli*, *Klebsiella pneumoniae*, and *Pseudomonas aeruginosa*.

(B) The combination of CDCA and imipenem, evaluated against four G^+ bacteria, *Enterococcus faecium*, *Enterococcus faecium*, *Staphylococcus aureus*, and *Streptococcus pneumoniae*.

Table S1. Strains used in this study.

Strain name	Features	References
ATCC 43300	MRSA	Lab collection
ATCC 25923	MSSA	Lab collection
MU50	VISA	Lab collection
150907015	Antibiotics resistance: penicillin-G, oxacillin, amikacin	Clinical laboratory of Guanganmen Hospital
151010052	Antibiotics resistance: penicillin-G, oxacillin, amikacin	Clinical laboratory of Guanganmen Hospital
180410023	Antibiotics resistance: penicillin-G, oxacillin	Clinical laboratory of Guanganmen Hospital
180211022	Antibiotics resistance: penicillin-G, oxacillin, amikacin	Clinical laboratory of Guanganmen Hospital
180126042	Antibiotics resistance: penicillin-G, oxacillin	Clinical laboratory of Guanganmen Hospital
171213054	Antibiotics resistance: penicillin-G, oxacillin	Clinical laboratory of Guanganmen Hospital
171116025	Antibiotics resistance: penicillin-G, oxacillin	Clinical laboratory of Guanganmen Hospital
171031048	Antibiotics resistance: penicillin-G, oxacillin	Clinical laboratory of Guanganmen Hospital
170911060	Antibiotics resistance: penicillin-G, oxacillin	Clinical laboratory of Guanganmen Hospital
170807024	Antibiotics resistance: penicillin-G, oxacillin, amikacin	Clinical laboratory of Guanganmen Hospital
170714063	Antibiotics resistance: penicillin-G, oxacillin, amikacin	Clinical laboratory of Guanganmen Hospital
161019014	Antibiotics resistance: penicillin-G, oxacillin; Intermediate: amikacin	Clinical laboratory of Guanganmen Hospital
161008022	Antibiotics resistance: penicillin-G, oxacillin; Intermediate: amikacin	Clinical laboratory of Guanganmen Hospital
160919055	Antibiotics resistance: penicillin-G, oxacillin	Clinical laboratory of Guanganmen Hospital
151009009	Antibiotics resistance: penicillin-G, oxacillin, amikacin	Clinical laboratory of Guanganmen Hospital
151020006	Antibiotics resistance: penicillin-G, oxacillin, amikacin	Clinical laboratory of Guanganmen Hospital
160115091	Antibiotics resistance: penicillin-G, oxacillin, amikacin	Clinical laboratory of Guanganmen Hospital
160123012	Antibiotics resistance: penicillin-G, oxacillin	Clinical laboratory of Guanganmen Hospital
1611017	Antibiotics resistance: penicillin-G, oxacillin, amikacin	Clinical laboratory of Dongzhimen Hospital
1611078	Antibiotics resistance: penicillin-G, oxacillin	Clinical laboratory of Dongzhimen Hospital
1611099	Antibiotics resistance: penicillin-G, oxacillin, amikacin	Clinical laboratory of Dongzhimen Hospital
1610057	Antibiotics resistance: penicillin-G, oxacillin, amikacin	Clinical laboratory of Dongzhimen Hospital
1702123	Antibiotics resistance: penicillin-G, oxacillin, amikacin	Clinical laboratory of Dongzhimen Hospital
1703118	Antibiotics resistance: penicillin-G, oxacillin	Clinical laboratory of Dongzhimen Hospital
ATCC 25922	*Escherichia coli*	Lab collection
ATCC 27853	*Pseudomonas aeruginosa*	Lab collection
ATCC 4352	*Klebsiella Pneumoniae*	Lab collection
ATCC 22933	*Acinetobacter baumannii*	Lab collection
ATCC 29212	*Enterococcus faecalis*	Lab collection
ATCC 19434	*Enterococcus faecium*	Lab collection
ATCC 49619	*Streptococcus pneumoniae*	Lab collection

Table S2. CDCA-treated MRSA differential proteins in exponential growth phase.

Protein	Description	Gene	UP&DOWN
A0A0H3JRU9	Pyruvate carboxylase	*pycA*	down
Q6GC82	Inosine-5-monophosphate dehydrogenase	*guaB*	down
P99161	Transketolase	*tkt*	down
Q6GFV0	Putative dipeptidase SAR1836	*SAR1836*	down
A7WYT1	Pyridoxal 5-phosphate synthase subunit PdxS	*pdxS*	down
Q6GFZ6	Acetate kinase	*ackA*	down
P63871	Cysteine synthase	*cysK*	down
Q5HKH9	Formate acetyltransferase	*pflB*	down
Q6GAV6	Coenzyme A disulfide reductase	*cdr*	down
Q6GID1	Ornithine aminotransferase 2	*rocD2*	down
Q6GJB4	Probable branched-chain-amino-acid aminotransferase	*ilvE*	down
Q5HRM8	ATP-dependent Clp protease ATP-binding subunit ClpC	*clpC*	down
Q5HCV0	L-lactate dehydrogenase 2	*ldh2*	down
Q6GDH2	Clumping factor B	*clfB*	down
A7WXP7	L-lactate dehydrogenase 1	*ldh1*	down
P63513	Probable branched-chain-amino-acid aminotransferase	*ilvE*	down
Q2FXL6	Putative universal stress protein SAOUHSC_01819	*SAOUHSC_01819*	down
Q5HL19	Probable malate:quinone oxidoreductase 4	*mqo4*	down
Q7A742	Alcohol dehydrogenase	*adh*	down
Q6GKB1	Type-1 restriction enzyme R protein	*hsdR*	down
Q6GGY2	DegV domain-containing protein SAR1438	*SAR1438*	down
Q2FWX9	4,4-diaponeurosporen-aldehyde dehydrogenase	*aldH1*	down
Q6GI15	Phosphoribosylformylglycinamidine synthase subunit PurL	*purL*	down
Q6GFB8	Protein map	*map*	down
Q2G1C2	Teichoic acid ribitol-phosphate polymerase TarK	*tarK*	down
Q2YXC5	Glutamate racemase	*murI*	down
Q6GGW9	Alanine dehydrogenase 1	*ald1*	down
Q6GIR8	Histidinol-phosphate aminotransferase	*hisC*	down
Q2FWZ8	Bacterial non-heme ferritin	*ftnA*	down
Q6GI14	Amidophosphoribosyltransferase	*purF*	down
Q6GK53	Iron-sulfur cluster repair protein ScdA	*scdA*	down
Q99W83	Pyridoxal 5-phosphate synthase subunit PdxT	*pdxT*	down
Q5HRR5	Methionine--tRNA ligase	*metG*	down
Q6GH18	Phosphate-binding protein PstS	*pstS*	down
Q6GEY7	Cardiolipin synthase 2	*cls2*	down
Q6GB81	DegV domain-containing protein SAS0714	*SAS0714*	down
Q6GI16	Phosphoribosylformylglycinamidine synthase subunit PurQ	*purQ*	down

Q6GI18	Phosphoribosylaminoimidazole-succinocarboxamide synthase	*purC*	down
Q2YY67	L-threonine dehydratase catabolic TdcB	*tdcB*	down
P56740	Dihydroneopterin aldolase	*folB*	down
Q99VJ0	Thermonuclease	*nuc*	down
Q5HQW4	Thioredoxin reductase	*trxB*	down
Q2FZZ2	Methionine import ATP-binding protein MetN 2	*metN2*	down
P0A0N7	Ribosomal RNA large subunit methyltransferase H	*rlmH*	down
A5ISF7	30S ribosomal protein S15	*rpsO*	down
Q2YYA2	Ribonuclease Z	*rnz*	down
Q6GI30	Putative acetyltransferase SAR1027	*SAR1027*	down
Q6GI19	N5-carboxyaminoimidazole ribonucleotide synthase	*purK*	down
A0A0H3JUG6	Epoxyqueuosine reductase QueH	*queH*	down
Q2YVB1	N-acetylneuraminate lyase	*nanA*	down
Q6GEM5	Uncharacterized hydrolase SAR2294	*SAR2294*	down
Q6G816	Sodium-dependent dicarboxylate transporter SdcS	*sdcS*	down
Q6GAE1	Phosphoribosylglycinamide formyltransferase	*purN*	down
Q99SE4	UPF0340 protein SAV2114	*SAV2114*	down
O32421	Probable cell wall amidase LytH	*lytH*	down
Q6GJM1	UPF0753 protein SAR0453	*SAR0453*	down
Q2YX68	UPF0637 protein SAB0972c	*SAB0972c*	down
Q2YX52	Phosphoribosylformylglycinamidine cyclo-ligase	*purM*	down
Q99S97	Multidrug efflux pump SdrM	*sdrM*	down
Q2YV56	Transcriptional regulatory protein HptR	*hptR*	down
Q2FJJ9	Uncharacterized lipoprotein SAUSA300_0416	*SAUSA300_0416*	down
Q5HL85	Putative ribose uptake protein RbsU	*rbsU*	down
Q6G6I7	Uncharacterized protein SAS2374	*SAS2374*	down
Q6GI31	Bifunctional autolysin	*atl*	up
A8Z4B9	Chaperone protein DnaK	*dnaK*	up
Q6GES3	Glutamine--fructose-6-phosphate aminotransferase [isomerizing]	*glmS*	up
Q6GIK4	Clumping factor A	*clfA*	up
Q6GEV3	Putative aldehyde dehydrogenase	*SAR2210*	up
Q6GI01	Phosphoenolpyruvate-protein phosphotransferase	*ptsI*	up
Q99V14	Phosphoenolpyruvate-protein phosphotransferase	*ptsI*	up
Q6GHI9	Succinate--CoA ligase [ADP-forming] subunit alpha	*sucD*	up
Q6GIE7	Type II NADH:quinone oxidoreductase	*SAR0903*	up
Q6GGZ6	Dihydrolipoyllysine-residue succinyltransferase component of 2-oxoglutarate dehydrogenase complex	*odhB*	up
Q6GIA3	3-oxoacyl-[acyl-carrier-protein] synthase 2	*fabF*	up
Q2G0P0	50S ribosomal protein L1	*rplA*	up
Q6GEX5	UDP-N-acetylglucosamine 1-carboxyvinyltransferase 1	*murA1*	up
Q8NX10	Phosphate acyltransferase	*plsX*	up
Q6GE17	2,3-bisphosphoglycerate-dependent phosphoglycerate	*gpmA*	up

	mutase		
Q6GA75	Phenylalanine--tRNA ligase beta subunit	*pheT*	up
Q2YSF6	Epimerase family protein SAB0724c	*SAB0724c*	up
A8Z425	Cell cycle protein GpsB	*gpsB*	up
Q6GB45	Clumping factor A	*clfA*	up
Q5HNL1	Isocitrate dehydrogenase [NADP]	*icd*	up
Q99UA7	Aminoacyltransferase FemA	*femA*	up
Q6GEV1	UDP-N-acetylglucosamine 1-carboxyvinyltransferase 2	*murA2*	up
A6U089	3-oxoacyl-[acyl-carrier-protein] synthase 3	*fabH*	up
A5ISB7	Acyl carrier protein	*acpP*	up
Q6GGE2	Probable endonuclease 4	*nfo*	up
Q6G664	Oxygen-dependent choline dehydrogenase	*betA*	up
Q2FXZ3	Chaperone protein DnaJ	*dnaJ*	up
Q6GFH7	UPF0435 protein SAR1970	*SAR1970*	up
Q5HE93	ATP synthase subunit b	*atpF*	up
P95695	Capsular polysaccharide type 5 biosynthesis protein cap5A	*cap5A*	up
Q6GHQ2	UDP-N-acetylmuramoylalanine--D-glutamate ligase	*murD*	up
P0C1S0	HTH-type transcriptional regulator MgrA	*mgrA*	up
A7X5E9	50S ribosomal protein L14	*rplN*	up
Q5HM31	50S ribosomal protein L13	*rplM*	up
Q2YYL3	50S ribosomal protein L18	*rplR*	up
Q5HPY8	Carbamoyl-phosphate synthase large chain	*carB*	up
Q7A6L9	UPF0337 protein SA0772	*SA0772*	up
Q6GJG1	Hypoxanthine-guanine phosphoribosyltransferase	*hpt*	up
A8Z2R5	Aspartyl/glutamyl-tRNA(Asn/Gln) amidotransferase subunit C	*gatC*	up
Q7A0Y4	Regulatory protein MsrR	*msrR*	up
Q6GEA0	Lysostaphin resistance protein A	*lyrA*	up
Q2FXZ1	Protein GrpE	*grpE*	up
P0C600	6,7-dimethyl-8-ribityllumazine synthase	*ribH*	up
Q6G789	50S ribosomal protein L30	*rpmD*	up
Q2G2P5	Nickel-binding protein NikA	*nikA*	up
Q2YSG0	Probable cell division protein WhiA	*whiA*	up
Q6GK35	Glycyl-glycine endopeptidase LytM	*lytM*	up
Q2G2P8	ADP-dependent (S)-NAD(P)H-hydrate dehydratase	*nnrD*	up
Q6GEX9	Probable transglycosylase SceD	*sceD*	up
P0A059	Potassium-transporting ATPase KdpC subunit 2	*kdpC2*	up
A0A0H3JT43	Glycine cleavage system H-like protein	*SAV0324*	up
Q6GI27	Teichoic acid D-alanine hydrolase	*fmtA*	up
P60074	UPF0291 protein SAV1341	*SAV1341*	up
Q5HQ35	Phenylalanine--tRNA ligase beta subunit	*pheT*	up
Q6G6U7	Ferredoxin--NADP reductase	*SAS2264*	up
Q6GDM3	HTH-type transcriptional regulator SAR2658	*SAR2658*	up

A8Z334	50S ribosomal protein L36	*rpmJ*	up
Q2G1T3	Organic hydroperoxide resistance protein-like	*SAOUHSC_00831*	up
Q5HMZ0	10 kDa chaperonin	*groS*	up
Q8NXW9	Putative proline/betaine transporter	*proP*	up
P39853	Capsular polysaccharide biosynthesis protein CapD	*capD*	up
Q7A805	N-acetylmuramic acid 6-phosphate etherase	*murQ*	up
A6U223	50S ribosomal protein L33 3	*rpmG3*	up
Q6GH76	Homoserine kinase	*thrB*	up
A6TZ11	50S ribosomal protein L33 1	*rpmG1*	up
Q6GDN4	Glycosyl-4,4-diaponeurosporenoate acyltransferase	*crtO*	up
Q5HM15	50S ribosomal protein L18	*rplR*	up
Q2YYQ7	Urease subunit beta	*ureB*	up
A5IU61	DNA polymerase IV	*dinB*	up
Q5HQ10	Phospho-N-acetylmuramoyl-pentapeptide-transferase	*mraY*	up
A6QGZ7	UPF0398 protein NWMN_1357	*NWMN_1357*	up
Q6GJ90	Protein VraX	*vraX*	up
Q2YX16	Probable quinol oxidase subunit 3	*qoxC*	up
Q5HQV8	Epimerase family protein SERP0438	*SERP0438*	up
Q99TA0	Riboflavin biosynthesis protein RibBA	*ribBA*	up
Q6GF11	Protein SprT-like	*SAR2150*	up
Q8NYB7	Protein-ADP-ribose hydrolase	*MW0302*	up
Q99SN8	Uncharacterized leukocidin-like protein 1	*SAV2004*	up
Q6GH70	30S ribosomal protein S14	*rpsN*	up
Q2FDE7	Ribonuclease P protein component	*rnpA*	up
Q2YY57	Holliday junction resolvase RecU	*recU*	up
